# A Novel Model on Reinforce K-Means Using Location Division Model and Outlier of Initial Value for Lowering Data Cost

**DOI:** 10.3390/e22080902

**Published:** 2020-08-17

**Authors:** Se-Hoon Jung, Hansung Lee, Jun-Ho Huh

**Affiliations:** 1 School of Creative Convergence, Andong National University, Andong 36729, Korea; jungsh@anu.ac.kr; 2 School of Computer Engineering, Youngsan University, 288 Junam-Ro, Yangsan, Gyeongnam 50510, Korea; 3 Department of Data Informatics, (National) Korea Maritime and Ocean University, Busan 49112, Korea

**Keywords:** initial seed, K-means, outliers, location division, density data, python data analysis, data science, hybrid, data driven

## Abstract

Today, semi-structured and unstructured data are mainly collected and analyzed for data analysis applicable to various systems. Such data have a dense distribution of space and usually contain outliers and noise data. There have been ongoing research studies on clustering algorithms to classify such data (outliers and noise data). The K-means algorithm is one of the most investigated clustering algorithms. Researchers have pointed out a couple of problems such as processing clustering for the number of clusters, K, by an analyst through his or her random choices, producing biased results in data classification through the connection of nodes in dense data, and higher implementation costs and lower accuracy according to the selection models of the initial centroids. Most K-means researchers have pointed out the disadvantage of outliers belonging to external or other clusters instead of the concerned ones when K is big or small. Thus, the present study analyzed problems with the selection of initial centroids in the existing K-means algorithm and investigated a new K-means algorithm of selecting initial centroids. The present study proposed a method of cutting down clustering calculation costs by applying an initial center point approach based on space division and outliers so that no objects would be subordinate to the initial cluster center for dependence lower from the initial cluster center. Since data containing outliers could lead to inappropriate results when they are reflected in the choice of a center point of a cluster, the study proposed an algorithm to minimize the error rates of outliers based on an improved algorithm for space division and distance measurement. The performance experiment results of the proposed algorithm show that it lowered the execution costs by about 13–14% compared with those of previous studies when there was an increase in the volume of clustering data or the number of clusters. It also recorded a lower frequency of outliers, a lower effectiveness index, which assesses performance deterioration with outliers, and a reduction of outliers by about 60%.

## 1. Introduction

There have been active efforts to create a new industry or increase the quality of medicine with data accumulated in the field of healthcare around the globe, and these efforts are contributing as important elements in the era of Smart Revolution [1,2,3]. One of the core elements to lead the era of smart revolution is data, whose production and spread have been developing at an alarming rate. There are active research studies on data mining, which involves collecting large volumes of data across various fields thanks to the advancement of information technologies, searching meanings, patterns, relations, and rules in collected data, and extracting useful knowledge by modeling data [4,5,6]. Data analysis and machine learning are categorized according to supervised and unsupervised learning in terms of data analysis [7,8,9]. In particular, unsupervised learning is a model of estimating relations with no labels in input data and with no training data. Representative algorithms of unsupervised learning are clustering and connection analysis [10,11,12]. Clustering is a technique of forming clusters based on similarity or dissimilarity (distance) among objects, examining the characteristics of clusters that have been formed, and analyzing multivariate data relations inherent between clusters [13,14,15,16,17]. In classification and clustering that is a basic algorithm of unsupervised learning, the K-means is most extensively used for its advantage of lowering execution costs [18,19,20,21,22,23,24,25]. And the K-means is a basic algorithm of unsupervised learning, and it is easily to development compared with other unsupervised learning. Nevertheless, K-means is completely dependent on the centroid of the initial clustering, whose selection causes widely differences in the execution time of clustering–repetition and the clustering results. In the K-means investigated in existing studies, the user arbitrarily determines the number of clusters to categorize initial data, and it leads to classification costs [26,27,28,29]. As data become more and more diverse in form and bigger and bigger in size, the fast execution that is the advantage of K-means cannot be maximized in the era of big-data [30,31,32,33,34]. When initial data sizes are as large as data, the methods of determining the number of clusters based on K-means such as the user’s will and the heuristic approach become a fundamental cause of lower clustering performance. There is a need for research studies on the methods of clustering that are appropriate for data mining. Many of the previous studies on the K-means algorithm selected initial centroids arbitrarily, measured the similarity between selected objects and others, and assigned K, the number of clusters [35,36,37]. Most K-means researchers [38,39,40,41,42,43,44,45] have pointed out the disadvantage of outliers (data out of normal scope) belonging to external or other clusters instead of the concerned ones when K is big or small [29]. Thus, this study set out to analyze problems with the selection of initial centroids in the old K-means algorithm and investigate an algorithm of selecting initial centroids for new K-means. There is a lot of study on algorithms in the model of selecting initial centroids through outliers and the division of space to generate ideal clustering results according to the selection of initial centroids. The proposed algorithm of selecting initial centroids would carry out clustering by choosing the objects of outliers reflecting negative impacts on clustering outcomes as initial cluster centroids. In the selection of outlier objects, the ones within the scope of $P\left(\bar{X}<x_{kl}\right)=\pm 0.95$ in the standard normal distribution of clustering objects would be used as initial centroids. Temporary cluster centroids would be selected in the set of outlier objects extracted from the standard normal distribution. Initial cluster centroids were selected from objects with low similarity and density distribution with other objects, and initial clusters were fragmented into two divided spaces (to secure two centroids). Once two centroids were secured, the objects with low similarity and the density from them would be selected as new cluster centroids. The two divided spaces would further fragmented into three divided spaces (to secure three centroids). This process was repeated to expand the number of clusters through the division of space. The algorithm of space division would be terminated when it matched the number of clusters designated by the user. The proposed algorithm would be compared and assessed with the old K-means algorithm according to various scales based on the datasets used in data analysis to check its superior performance.

## 2. Related Research

### 2.1. K-Means Algorithm

The proposed K-means algorithm in this study can be useful when grouping input data into K clusters for unsupervised learning. What makes this clustering algorithm different from the conventional algorithms often used for the supervised learning is that the weight vectors are updated at the same time only after entire input vector entry has been completed, rather than repeating the update process each time a vector enters. The terms of cluster classification are as follows: the distance between clusters, dissimilarity of clusters, and minimized level of cost functions becoming similar or equal. With this classification model (algorithm), the data objects in the same cluster become more similar compared to the data objects in the other clusters. Meanwhile, the individual centroid of each cluster and the sum of squares of distances between data objects are used to create a cost function for the minimization task that will be repeated to classify and assign every data object to a certain cluster [5,14,15,16,17,46,47,48,49,50,51,52]. The K-means algorithm is a clustering technique to classify input data into K clusters based on unsupervised learning. Unlike supervised learning, which updates weight vectors every time a vector is entered, the K-means algorithm updates weight vectors simultaneously after all the input vectors are entered. The criteria of clustering classification are the distance between clusters, dissimilarity among clusters, and minimization of the same cost functions. The similarity between data objects increases within the same clusters, while the similarity to data objects in other clusters decreases. The algorithm performs clustering by setting the centroid of each cluster and the sum of squares between data objects and distance as cost functions and minimizing the cost function values to repeat the cluster classification of each data object. By calculating the sum of each length of an input vector from the centroid of a cluster and then measuring the distance between individual centroids, an Intra-Cluster Distance (IntraCD) can be estimated, whereas Inter-Cluster Distance (ICD) represents the distance (weight vector) between two clusters. In Equation (1), the sum of all the ICDs calculated for each pair has been subtracted from the sum of all the IntraCDs to compute an error. In the equation, both β and γ represent weighted values. The K-means algorithm repeats epochs until the errors no longer decrease or the cluster composition no longer changes. Errors are checked by measuring within-cluster and between-cluster distances. In general, β and γ are set at 0.9 and 0.1, respectively, in an experiment. In this paper, we set weights of 0.9 and 0.1 in an experiment with the K-means algorithm.

(1) $$\mathrm{Error}=\;\beta \overset{k}{\underset{i=0}{\sum }}\left(Intra\;CD\right)-\;\gamma \overset{k}{\underset{i=0}{\sum }}\left(ICD\right)$$

The early K-means clustering algorithm (Lloyd’s 1957) is one of the most widely used algorithms even today for its speed and simplicity, but the initial clustering algorithm based on the greedy approach has been pointed out to have a couple of issues, including sensitivity to initialization according to classification data and a deterioration of classification performance according to initial center selection. Thus, the basic K-means algorithm has been partially altered with various research studies done according to the initial center selection of classification data. Ref. [53] proposed a series of refined initial starting points for K-means clustering algorithms with datasets divided into small random subsamples. K-means clustering and the minimum error values of these subsamples were calculated and used as initial clustering centers. Ref. [54] introduced a clustering center initialization algorithm (CCIA) to calculate initial centers for K-means clustering and conducted research based on the similarity of data patterns. Ref. [55] introduced a method of obtaining initial centroids for K-means clustering and proposed an algorithm combined with K-means and hierarchy algorithms. In its early stage, K-means clustering was applied through random initialization. The algorithm performed hierarchical clustering after the convergence of initial clustering results to get better results, being usually used for bigger numbers of clusters or attributes. Ref. [56] proposed an algorithm of calculating initial cluster centers for the K-means algorithm. The proposed algorithm chose two major axes with an ‘f’ variable and calculated the initial central axes based on these axes. The two axes were chosen for smaller correlations between variables, and at the same time, they had a problem of a high deviation coefficient. Ref. [57] defined the closest neighbor pair and proposed four hypotheses for it. These hypotheses were based on the center initialization method of the K-means algorithm for datasets containing two clusters. Ref. [58] proposed a revised K-means algorithm making use of proper centers. The researchers maintained that this algorithm should reduce the number of repetitions. Ref. [59] proposed weighted values for the distributed data of K-means algorithm, assigning weighted values to clusters related to dispersion and optimizing the initialization issue. Ref. [60] proposed an algorithm to determine the initial center of K-means clustering for labeling and non-labeling datasets. Ref. [61] proposed a new initialization process for K-means clustering based on the binary search technique. In this process, the minimum and maximum values of each attribute were selected after the application of binary search attributes to the calculation of initial cluster centers. Ref. [62] proposed an initialization algorithm for K-means clustering based on hybrid distance, combining Euclidean distance and density-based distance.

### 2.2. Clustering K Value in Non-Labeling Data

The simplest approach to selecting the appropriate K without domain knowledge is an empirical model, i.e., increasing the initial cluster number gradually. Nonetheless, this approach requires large computation power and considerable cost as the size of the dataset increases. To solve this problem, a large number of previous studies focused on how to achieve clustering with high accuracy without domain knowledge based on the finite mixture model. In a study that proposed rival penalized controlled competitive learning for data clustering without knowing the exact number of clusters, the centers of two parties, a winner and a competitor, were considered; the winner was the closest center, and the competitor was the second closest center to each data point. This model lets the competitor choose the appropriate center by utilizing an unlearning rate parameter. Competitive learning may perform data clustering without knowing the initial number of clusters, but it was sensitive to the unlearning rate of the competitor selected in advance, and it could not guarantee the optimized model. Unlike existing K-means algorithms, the proposed K-means algorithm consists of two major steps. In the first step, the K-means algorithm performs initial clustering followed by pre-processing for assigning a seed point to each cluster. Finally, the assigned seed point is adjusted to be minimized. In this step, a cluster whose number of data is larger is selected as the priority, and a cluster whose number of data is smaller becomes the center of the empty cluster; thus, it is excluded from the priority candidate. As such, K-means selects the initial center point, which has no domain knowledge. The K-means++ algorithm has proposed an initialization process for the K-means clustering algorithm. It was based on choosing a random starting center with a specific probability, and it was called the K-means++ algorithm. The K-means++ algorithm is provided to solve the problem of unstable learning results of clustering when it is terminated. The K-means++ algorithm proposed for this adjusts the sampling probability distribution. In other words, a point whose distance is greater than that of the previously selected point is selected with higher probability in the step that selects the initial point. Accordingly, the initial point is selected as the point whose distance is greater than that of the previously selected point. Note that the K-means ++ algorithm cannot solve the cluster locking problem, hence the need to have a strategy for selecting the initial point to solve the clustering locking problem.

### 2.3. Clustering Initial Approach Algorithm

The initialization approach for K-means clustering is to select the number of clusters by changing it from an initial value to a set value and checking the clustering results. Then, a heuristic approach is often used to determine the optimal number of clusters. Although it would be possible to select the value that minimizes the resulting value of either Akaike Information Criterion (AIC) or Bayesian Inference Criterion (BIC) as a centroid value of a cluster when a clustering methodology based on probabilistic structure was used, it is not easy to determine the optimized centroid value otherwise. For the selection of an initial value for K-means clustering, which is a non-hierarchical model, the sum of the squared distances tends to decrease in most cases as the initial K-value increases. For this reason, there is a model of calculating the sum of the squared distances while increasing the K-value and selecting the one whose decrement in sum was reduced from the previous sum when K-1, if there is any. Another type of model, i.e., hierarchical clustering, does not require setting the number of clusters in the early stage but has to select it when distinguishing the clusters in the end. Although a variety of models can be utilized for this type of clustering when determining an adequate number of clusters, a model that searches the optimal number through the visualization of objects is used widely. The Macqueen Approach, Kaufman Approach, Max–Min model, and K-means++ model are being used these days along with some types of heuristic approaches, but the other models are also being researched as well. Some of the typical research works on selecting an initial K-value and centroid value for K-means clustering are described below. Macqueen Approach K-means (MAK) presents a model usually used in researches on the utilization of the K-means clustering algorithm [63]. The user arbitrarily selects K, the initial number of clusters, from the initial objects for clustering. K can be randomly selected from the entire objects. K is assigned to the closest cluster according to the measurement of distance from the cluster centroid to all of the objects. The objects assigned to a cluster will be finally used for reassignment to new centroids. The algorithm will be terminated when centroids are measured under the threshold value set by the user. Kaufman Approach K-means (KAK) introduces an algorithm to supplement the rising costs of measurement due to the calculation of distance from all of the objects, which had been pointed out as a problem with MAK [64]. The algorithm is faster in measurement than MAK. This algorithm determines the centroids of all the objects distributed as initial centroids. The distance is measured from an object of the entire group to the initial cluster value. When a selected object has a distance from the initial value over the threshold value set by the user and has a certain number of objects nearby or more, it will be determined as a cluster centroid. The algorithm would repeat the process until the initial K value matches the selected outcome until termination. However, the approaches of MAK and KAK to initialization have problems of selecting initial values arbitrarily and choosing initial values according to certain conditions and rules. Max–Min Approach K-means (MMK) selects an object from the entire group and determines it as the first initial value and measures its distance from the other objects [64]. The object with the longest distance from the first initial value is assigned as the second initial value. The distance from the other objects will be calculated based on the first and second initial values. Since two initial values are needed to calculate distance, the sets of distance pairs will be measured to save two measurements. The initial value of distance pairs from the measured set of distance pairs will be defined as the distance measured between the concerned object and two values. The observed value with the maximum distance measurement will be determined as the third initial value, which will have the longest distance from the earlier two initial values assigned. The algorithm will continue until the initial values of K are all met through the repeated process. K-means++ (KMP) selects an object from the entire group arbitrarily and assigns it as the first initial value [65]. Distance is measured from the first initial value to the entire objects. Each measurement will be converted into squared distance and divided by the squared addition of distance of all the objects to calculate the probability of selecting the second initial value. Starting with the third initial value, the distance from the other objects will be calculated in the same model as MMK. Then, the probability calculation will be repeated to assign initial values.

## 3. Proposed Reinforcement K-Means Algorithm

### 3.1. Overview of Proposed Reinforcement K-Means Algorithm

The initialization approach model of K-means clustering is a model of selecting the number of clusters, which is changed from the initial value to the setting value to check the cluster results. After this, the heuristic approach model that determines the optimal number of clusters is largely utilized. For clustering methodologies based on a probabilistic structure, the results that minimize the values of Akaike Information Criterion (AIC) or Bayesian Inference Criterion (BIC) may be selected as a center point of the cluster. However, for clustering methodologies that are not based on a probabilistic structure, it is not easy to find a model that determines the optimized center point of the cluster. For the selection of the initial value of K-means clustering, which is a non-hierarchical model, the sum of squares of distances tends to decrease in general as the initial center K value increases. As a result of this, in some models, the sum of squares of distances is calculated while increasing the initial center K value, and the cluster center value is selected if there is a K value that reduces the decrement of sum of squares of distances more than the previous sum of squares of distances when K-1. For other clustering models such as hierarchical clustering methodology, the number of clusters need not be determined initially. Nonetheless, the number of clusters must be selected when the cluster is finally classified. Although various models may be utilized to determine the appropriate number of clusters in hierarchical clustering, a model of searching for the optimized number of clusters through object visualization is ideal. There is a need for a scale to assess whether analysis data are similar or dissimilar in the clustering model not based on the probability model of data. Clustering is usually done with dis-similarity (or distance) rather than similarity. The model of determining K, the number of clusters, is critical for optimal clustering. The present study used K, the optimal number of clusters, to set a temporary scope through the principal component analysis of input data. The scope of principal components to minimize the sum of distance squares within the set scope was appointed as the final number of clusters, i.e., K. 

### 3.2. Selection of Proposed Reinforce K-Means Initial Centroid Approach

#### 3.2.1. Outliers Generating Condition

**Figure 1:**
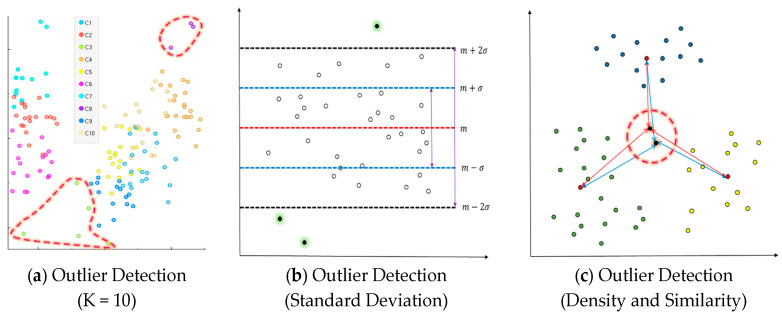
Clustering outliers generating condition.

An outlier is an element influencing the classification outcomes in a data classification algorithm. Previous studies on outliers uniformly tended to increase the accuracy of classification algorithms based on distance measurement and lower the number of outliers. Their approach was not to resolve issues with the number of outliers fundamentally, but to lower the number of outliers while increasing classification outcomes. The present study proposed a method of measuring outliers predicted in a way of reducing them in advance and making use of them to determine an initial centroid of a cluster. There are three major conditions required to generate outliers: first, outliers will happen in most cases of high K, the number of clusters is set high before generating an initial value. Figure 1a shows an example of outliers in case of high K. The example had outliers discovered in two clusters in a case of clustering the data with 10 initial clusters. Secondly, the probability of outliers will rise according to the dispersion of input data. The data of K-means clustering can be regarded as good clustering when there is a density around the centroid of a cluster and the overall density of data is high. In particular, dispersion is the degree of data being scattered. When its measurement is smaller, the variate is located closer to the mean. When the standard deviation based on the dispersion value is over the threshold value, certain objects will often become farther from the center of variates. For instance, the probability of outliers will be high when the standard deviation is over the threshold value based on the dispersion value of the entire data with clustering completed. Figure 1b presents an example of outliers according to dispersion. Thirdly, the probability of outliers is also high when some of the entire objects have lower density and similarity than others. Similarity between objects is measured with squared Euclidean distance. When the mean distance measurement from all the objects except for the initial cluster centroid is higher than the mean of each cluster, the probability of outliers increases. When the distance measurement from neighboring objects is high on average, the density will be low, which means a higher probability of outliers. Figure 1c introduces an example of judging distance measurements based on density and similarity.

#### 3.2.2. Algorithm to Determine the Initial Center with Outliers and Space Division

**Remark 1.**  *This study proposed an algorithm to select an initial center with **[Consideration 1, solving the outliers of given objects.] and [Consideration 2, maximizing the efficiency of the initial value K selection by using space and cluster distance.]** that were necessary and sufficient conditions of outliers. The proposed algorithm was designed to select individuals that could be outliers among input data and choose one as a temporary centroid of a cluster. The initial centroid of clustering is chosen among outlier-predicting individuals with low inter-individual similarity and density based on standard normal distribution. An initial cluster is divided into two spaces (securing two centroid). After selecting two centroid of a cluster, an additional individual with low similarity and density to the two centroid will be chosen as a new centroid of a cluster with two spaces further divided into three spaces (securing three centroid). As this process is repeated, the number of clusters will grow through spatial division. Once the number matches the optimal number of clusters, the spatial division algorithm will be completed. Clustering will proceed for the remaining individuals. Below is the definition of details to implement a clustering algorithm based on outliers and spatial division*.

**Assumption 1.**  *All of the input data can provide all sorts of information to set the initial centroid of repeating clusters, and the similarity and density distribution of objects can be used along with the location of standard normal distribution to remove anticipated outliers. The entire between-data data were altered with continuous probability distribution based on a proximity scale between data according to random data (outermost point in distance measurement among multidimensional data) to choose the initial center point of clustering. In a proximity scale, a log-likelihood value between random data and each data was divided by the log-likelihood value of a random data model. A proximity scale is smaller than 1 in most cases and can be defined as the "degree of explaining distance components between data with connection components between data". In other words, when a proximity scale value is close to 1, connection components between two data will be excluded from clustering as outlier candidates for connectivity and clustering. When it is close to 0, it can be chosen as an initial center point*.

**Theorem 1.**  *Equation (2) is a formula to define the objects of observed values. When selecting the centroid (*$m_{k}$*) of the first cluster (*$C_{1}$) of all the input vector data, the user can define it with the objects of observed values in the scope based on the standard normal distribution ($N_{\mu ,\;\sigma^{2}}\left(x_{k},y_{k}\right)$*) of*$P\left(\bar{X}\geq x_{k},y_{k}\right)=\pm 0.95$.

(2) $$N_{\mu ,\;\sigma^{2}}\left(x_{k},y_{k}\right)=\left(\frac{1}{\sigma \sqrt{2\pi }}e^{-\frac{{\left(x-\mu \right)}^{2}}{2\sigma^{2}}}\right)*\left(\;\frac{1}{\sigma \sqrt{2\pi }}e^{-\frac{{\left(y-\mu \right)}^{2}}{2\sigma^{2}}}\right)$$

$$N_{\mu ,\;\sigma^{2}}\left(x_{k},y_{k}\right)=\left(\frac{1}{\sigma }N\left(\frac{x-\mu }{\sigma }\right)\right)*\;\left(\frac{1}{\sigma }N\left(\frac{y-\mu }{\sigma }\right)\right)$$

**Figure 2:**
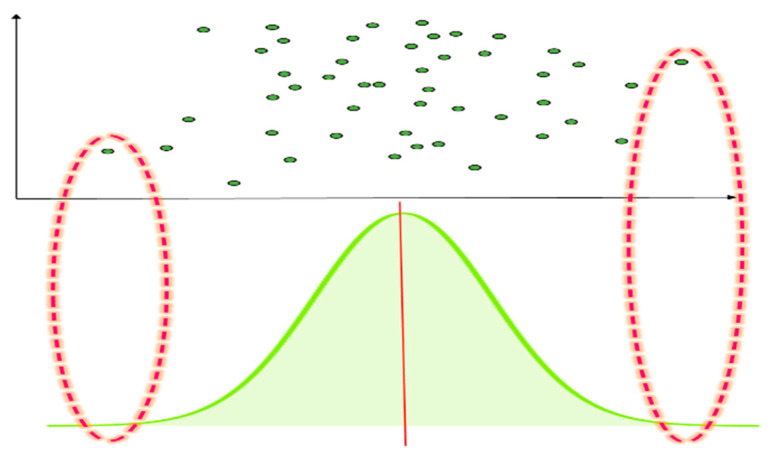
Example of standard normal distribution for input data (e.g., $P\left(\bar{X}<x_{kt}\right)=\pm 0.95$).

Figure 2 presents an example image of a standard normal distribution of input data. When the number of input example data is n, it will follow the standard normal distribution. When the objects are within ±0.95 of the standard normal distribution scope, the observed values will be assigned to $m_{k}$, the first cluster centroid.

**Proof of Theorem 1.**  $k^{2}$*or less will be an observed value to be an initial centroid of a cluster for each of randomly selected objects based on the application of basic standard normal distribution according to the algorithm defined in Theorem 1. The conditions of an observed value include*X*that is the set of input data,*μ*that is a mean, and that is standard deviation. When clustering proceeds with 1 or higher for K, the number of optimized clusters, it will satisfy Equation (3)* [66].

(3) $$P\left(\left|x-\mu \geq k\sigma \right|\right)\leq k^{2}$$

$$\begin{array}{c} P\left(\left|x-\mu \geq k\sigma \right|\right)=P{\left(x-\mu \right)}^{2}\geq k^{2}\sigma^{2}\\ =\frac{k^{2}\sigma^{2}}{E\left[{\left(x-\mu \right)}^{2}\right]}\\ =\;\frac{k^{2}\sigma^{2}}{\sigma^{2}}\\ =k^{2} \end{array}$$

□

**Theorem 2.**  *The formula to measure an object*$A_{k}$*distance will be defined as Equation (4) to measure similarity and density among objects when there is one object or more in the observed values of centroids defined in Theorem 1. The mean distance measurement between objects,*$AVG_{k}$*, will be defined as Equation (5)*.

(4) $$A_{k}=d\left(x_{ki},\;x_{li}\right)=\;\sum_{k=1}^{k}\sum_{i\in C_{1}}{\left(X_{i}-X_{k}\right)}^{2}$$

(5) $$AVG_{k}=\frac{1}{k}\sum_{k=1}^{k}\sum_{i\in C_{1}}{\left(X_{i}-X_{k}\right)}^{2}$$

**Figure 3:**
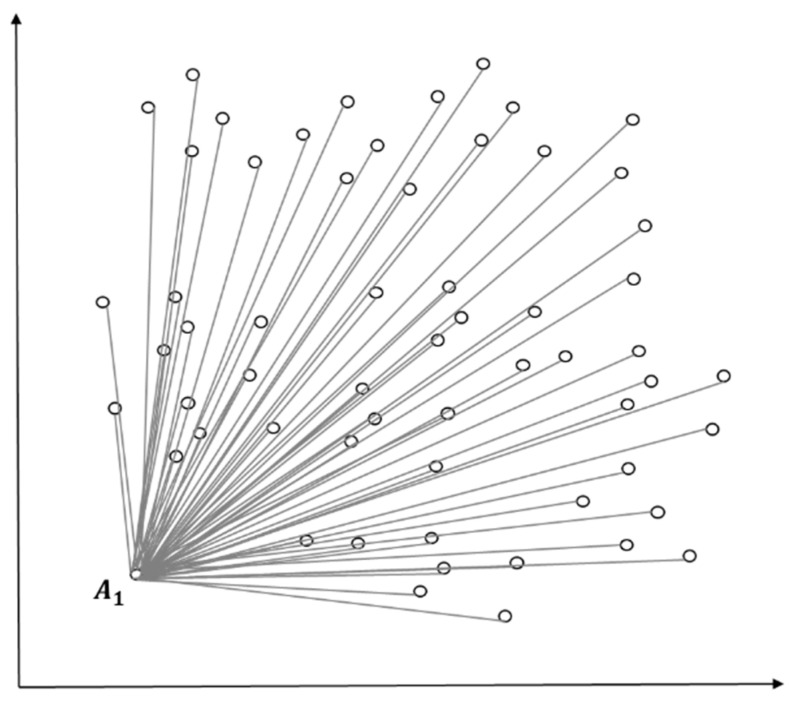
Distance measure of initial values (e.g., $A_{1}$).

**Figure 4:**
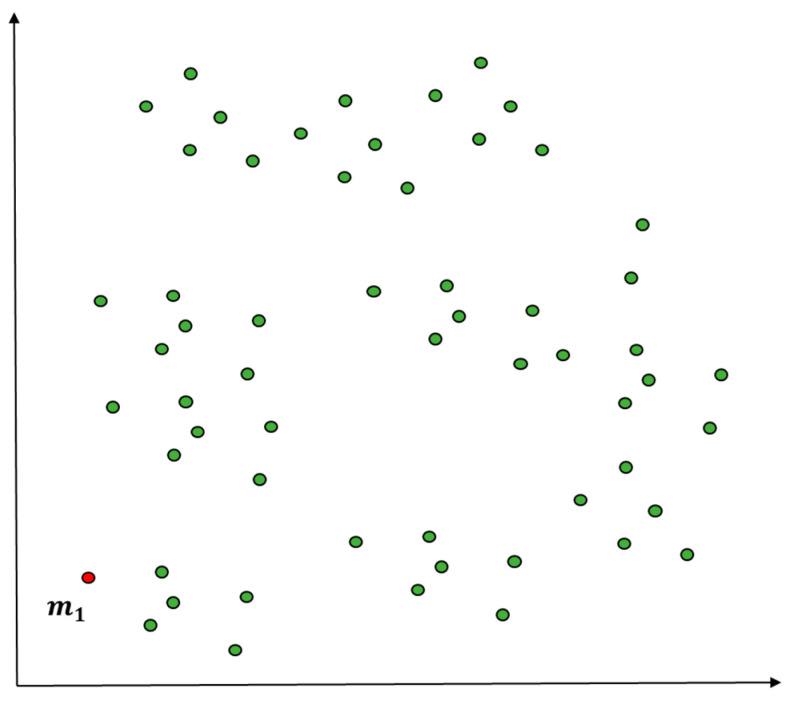
Initial value selection using distance measure with prediction outlier (K = 1).

**Figure 5:**
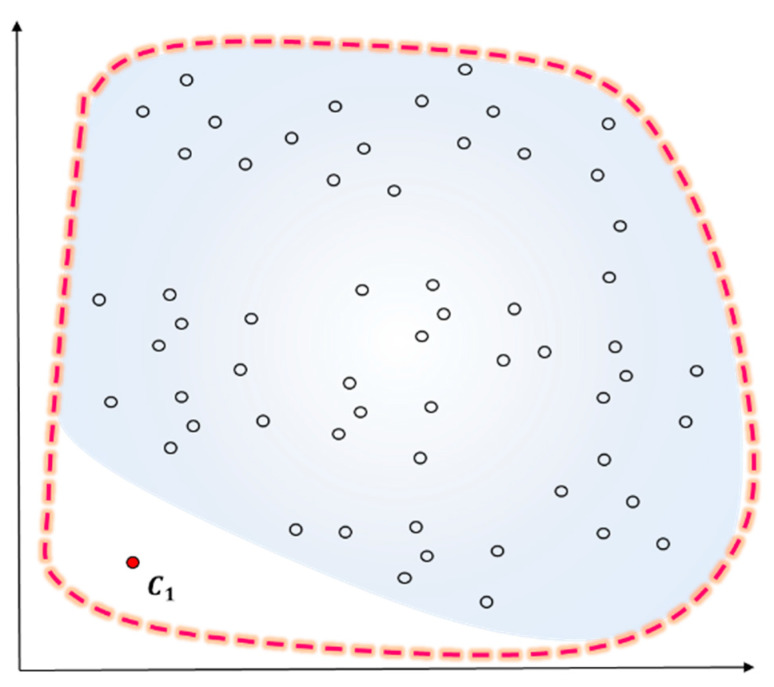
Assignment of initial cluster (e.g., $C_{1}$).

Figure 3 presents an example of using squared Euclidean distance to measure similarity and density between the entire objects and certain objects (predictive value according to standard normal distribution) when the entire initial objects are considered as a cluster. The measurement $A_{1k}$ is lower in similarity and density than other objects and then assigned as the initial value of the first cluster as in Figure 4. In Figure 4, clustering happens at $m_{1}$, the centroid of the initial cluster $C_{1}$, to select outliers according to the standard normal distribution as in Figure 5. The entire data that have been entered will be grouped into a cluster according to the initial conditions.

**Theorem 3.**  *Distance between objects*$A_{k}$* will be measured for all the objects*$x_{1}$*except for the centroids according to*$m_{1}$*, the centroid of the initial cluster*$C_{1}$*. The object with the maximum measurement will be assigned to*$C_{2}$*, the centroid of the second cluster. Equation (6) defines the way of assignment to the second cluster*.

(6) $$C_{2}\left(m_{2}\right)=\alpha_{i}\leftarrow \underset{1\leq i\leq n}{\max}\left(A_{k}\right)$$

$$C_{2}\left(m_{2}\right)=\;\underset{1\leq i\leq n}{\max}\left(A_{k}\right)∥x_{i}-m_{1}∥$$

$$C_{2}\left(m_{2}\right)=∥\alpha_{i}-m_{1}∥$$

**Figure 6:**
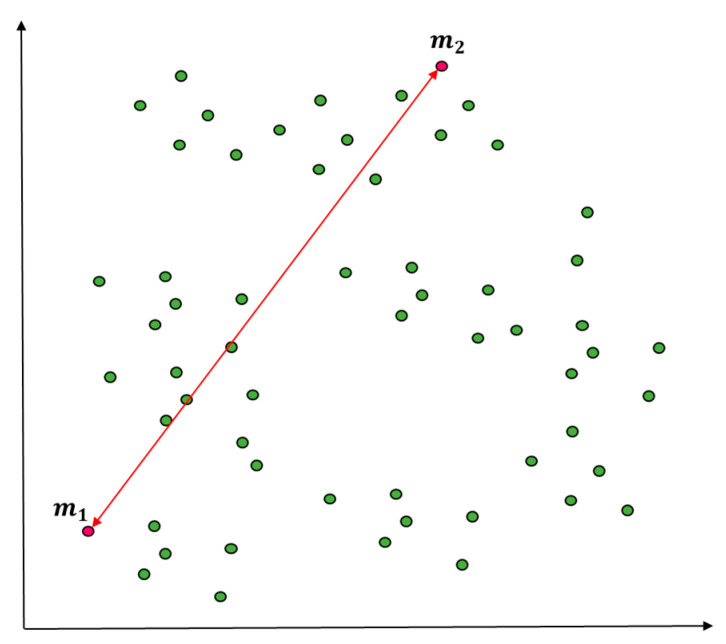
Centroid value selection using distance measure with space division (K = 2).

**Figure 7:**
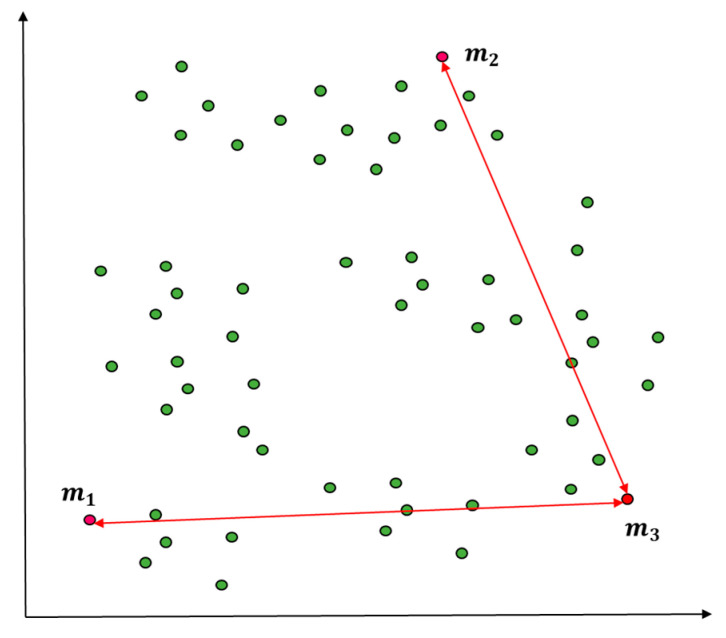
Centroid value selection using distance measure with space division (K = 3).

Figure 6 and Figure 7 show the centroid of the first cluster $C_{1}$ (Green Color) by applying Equation (6). Of the measurement data of the observed value sets, the object with the maximum distance from $m_{1}$ will be assigned to $m_{2}$, the centroid of the second cluster $C_{2}$.

**Theorem 4.**  *Distance between objects for the remaining objects *$x_{i}$* will be measured as follows except for*$m_{1}$*, the centroid of the initial cluster*$C_{1}$*, and*$m_{2}$*, the centroid of the second cluster*$C_{2}$*, through the two clusters defined in Theorem 3: the sets of distance pairs from initial centroids*$m_{1}$*,*$m_{2}$*will be distinguished, and the set values of distance pairs will be measured. Of the sets, the object with the maximum value will be assigned to*$m_{3}$*, the centroid of the third cluster*$C_{3}$.

(7) $$CA_{i}=\max∥x_{j}-m_{1}∥,\;∥x_{j}-m_{2}∥,\;1\leq j\leq n$$

(8) $$C_{3}\left(m_{3}\right)=x_{i}\leftarrow \underset{1\leq j\leq n}{\max}\left(CA_{j}\right)=CA_{i}$$

In Figure 7, the distance from the remaining objects $m_{3}$ …$\;m_{k}$ is measured after defining $m_{1}$ and $m_{2}$ as the centroids of two clusters $C_{1}$ and $C_{2}$ according to Theorem 4. When the old cluster centroids are $m_{1}$ and $m_{1}$ = (2, 1) and $m_{2}$ = (8, 10), for instance, the measurement of distance from a certain object $m_{i}$ = (12, 3) can be summarized as Vector X = 14 and Vector Y = 9. Based on these vector length results, an object with maximum length will be assigned to $m_{3}$, the centroid of $C_{3}$.

**Figure 8:**
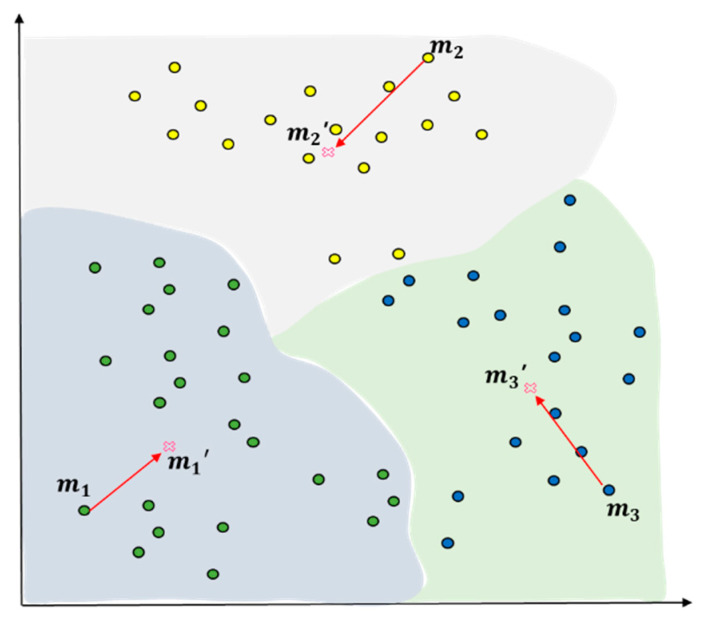
Finish of centroid value select using distance measure with space division (K = 3).

In the final stage, $C_{k}$, when the number of newly generated clusters matches K, the number of initial clusters for clustering, the initialization algorithm will be ended with the remaining observed values moved or assigned to the old clusters. The centroids of each cluster, $m_{1},\;m_{2},\;m_{3}\ldots ,\;m_{k}$, will be recalculated and assigned as new centroids. Figure 8 shows the outcomes of the final clustering when the entire number of objects was 57 with K = 3.

### 3.3. Proposed Reinforcement K-Means (PKM) Initial Approach Algorithm

Algorithm 1 shows the entire algorithm of the system to which the classification technique of data proposed in the study was applied [5,66,67]. Most of data for classification is basically multi-dimensional and multi-variate with individuals connected to one another. Many previous studies investigated approaches to the costs of data classification and the initial centroid of a cluster. The realization of a clustering algorithm requires the following necessary conditions: first, methodology for the clustering of data includes methods to measure space and distance. The given method of dividing individual spaces should be utilized along with the method of measuring distance between individuals and clusters especially for the assignment of initial cluster values; secondly, there is a need to secure an optimal central value early for a multi-dimensional individual. The reduction of dimensions should be considered as an optimal way of clustering for multi-dimensional individuals. If connections between clusters are minimized through the reduction of dimensions, it can increase the accuracy of selecting an initial cluster; and finally, the outliers of given individuals should be reduced to the minimum. When an outlier is chosen for the central value of a cluster, it can result in improper outcomes. Error rates of outliers should be minimized with an algorithm of improved spatial division and distance measurement. Along with these three necessary conditions, the present study proposed PKM based on two methods to improve the efficient clustering of multi-dimensional input data, which was its main purpose. First, it proposed a technique of selecting K, the number of clusters [66] through principal component analysis for the reduction of multiple dimensions. And secondly, it proposed a technique of approaching the center of K-means initialization in non-hierarchical clustering through outliers and spatial division. 

The proposed algorithm had the following flow: the stage of determining K, the optimal cluster, performs clustering for data and selects K, the optimal number of clusters. The pre-processing stage conducts principal component analysis by changing the multi-dimensional data of characteristic vector linearly and selects K, the optimal number of clusters emerging from the scope of principal component analysis. K, the optimal number of clusters, is determined based on differences in the scope of principal component analysis [66]. At the stage of determining an initial central model, an initial centroid of clustering is selected through outlier objects and spatial division based on K defined at the stage of determining K. An object predicted based on standard normal distribution is selected as an initial centroid. Distance is measured between the selected object of initial centroid and all the objects. The object of maximum value among them is selected as the second centroid of clustering. This process will be repeated until K, the optimal number of clusters, is satisfied. Each data object is determined with a cluster of the highest similarity level to the centroid of a cluster.
**Algorithm 1** Reinforcement Clustering using PCA and Initial Centroid Subspace      ***Data**: Non-Labeling Dataset*     ***Output**: Data by Cluster* ***Input:***       *Training set*
$x^{\left(1\right)}$*,*$x^{\left(2\right)}$*,*$x^{\left(3\right)}$*, …*$x^{\left(n\right)}$
     *where*
$x^{\left(i\right)}\in \;{\mathbb{R}}^{n}$
*(drop*
$x^{1}$
*= 1 by convention)*     ***repeat***
*each*
$SK_{i,}\;\alpha \in \left\{1,\;2,\;\ldots ,\;n\right\},$
***do***
     ***for* ***each temporary*
$\;initial\;centroid\left(m_{k}\right)<\;SK_{i-1\;,}$*, **do***            *calculation from each data to cluster centroid(cohension),*$A_{k}$            $A_{k}=\;\sum_{k=1}^{k}\sum_{i\in C_{k}}{\left(x_{i}-m_{k}\right)}^{2}$            *calculation from each data to cluster centroid(separation),*
$B_{k}$            $B_{k}=\;min{\left\{\left(x_{i}-m_{k}\right)\right\}}^{2}$            *assign the initial centroid number, S(K)*            $S\left(K\right)=\;\frac{1}{N}\frac{B_{k}-A_{k}}{max\left(A_{k},\;B_{k}\right)}$   ***end***     ***if** Selection -> Clustering number K*      ***then***        ***for***
*check of each vector data,*
$\alpha \in c_{k}$                                    *each*
$c_{k}$*=*$N_{\mu ,\sigma^{2}}\left(x_{k},y_{k}\right)=\left(\frac{1}{\sigma \sqrt{2\pi }}e^{-\frac{{\left(x-\mu \right)}^{2}}{2\sigma^{2}}}\right)*\left(\frac{1}{\sigma \sqrt{2\pi }}e^{-\frac{{\left(y-\mu \right)}^{2}}{2\sigma^{2}}}\right)$        ***if***
$P\left(\bar{X}\geq x_{k},\;y_{k}\right)=\pm 0.95$*>*
$c_{k}$*, which is the initial centroid*
          ***if** when there is two object distributed*
            ***then***
*assign the object to*
$m_{1}$*, the centroid of*
$C_{1}$*, the first cluster*          ***else***            ***then***
*assign the object whose two vectors record the biggest length first to*
m*, the centroid of*
$C_{1}$, the first cluster               ***for***
*check of each vector data,*
$\alpha \in c_{i}$                   $C_{2}\left(m_{2}\right)=a_{i}\leftarrow \underset{1\leq i\leq n}{max}\left(A_{k}\right)=\;\underset{1\leq i\leq n}{max}∥x_{i}-m_{1}∥=∥\alpha_{i}-m_{1}∥$                  ***for***
*check of each vector data,*
$\alpha \in c_{j}$                ***if***
*(of the maximum measurements)*                       ***then***      $CA_{i}=max\;∥x_{j}-m_{1}∥,\;∥x_{j}-m_{1}∥$     ***until***
*each cluster centroid.*                  ***end***               ***end***            ***end***       ***end*** ***end main***



Principal components were identified until the point where a certain value was maintained to explain the entire data through principal component analysis for the entire input data objects. Based on the principal components identified through principal component analysis, the center point division method was applied to $C_{ki}$, the number of random clusters, and $n_{k}$, the number of center points randomly selected. Random cluster index vectors were used to measure $m_{k}$, the center point of each initial cluster. k  is a random maximum value. The number of clusters was set at a maximum value in an experiment. It was set at 100 in my study. A minimum value was calculated with $A_{k}$, the addition of each object’s distance square from the center point of each divided area. $B_{k}$ was obtained, which was the minimum value of mean distance between random center points in a cluster and the objects included in an external cluster. S(K), cluster dissimilarity with a maximum value, was processed as $C_{k}$, the number of clusters K, based on differences between $A_{k}$ of separation, which uses the mean distance between objects included in a different cluster and the other objects, and $B_{k}$ of cohesion, which uses the mean distance between an object in a cluster and one in an external cluster. S(K) is between −1 and 1. As it is closer to 1, it is defined as an optimized number of clusters. The defined vector data was set with $C_{k}$, the number of clusters. Mean μ, standard deviation σ, and standard normal distribution $N_{\mu ,\sigma^{2}}\left(x_{k},y_{k}\right)$ were measured for all the vector data (α). A spatial quantile value was selected, which used the mean μ and standard deviation σ of vector data along with objects distributed in $P\left(\bar{X}\geq x_{k},\;y_{k}\right)=\pm 0.95$. When there was one object distributed, it was assigned to the center point $m_{1}$ of the first cluster $C_{1}$. When there were two objects or more distributed, the one with the maximum distance between two vectors was first assigned to the center point $m_{1}$ of the first cluster $C_{1}$. The distance between the other objects ($x_{i}$) and the center point $m_{1}$ of the first cluster $C_{1}$ was measured, and the object with maximum distance was assigned to the center point $m_{2}$ of the second cluster $C_{2}$. Except for $m_{1}$ and $m_{2}$, the center points of clusters $C_{1}$ and $C_{2}$, respectively, the remaining objects ($x_{i}$) were measured for distance as follows: distance pairs were measured with the center points of clusters, and a maximum value was measured with sets of distance pairs. The object with the highest maximum value measurements was assigned to $m_{3}$, the center point of the third cluster $C_{3}$.

## 4. Experiments and Performance Evaluation

### 4.1. Environments of Experiments and Performance Evaluation

The Proposed Reinforce K-means algorithm(PKM) proposed in the present study can be assessed and developed further with its counterparts in previous studies under the following conditions: Windows 10 64 bit, RAM 16 GB, Python as the language of development, and Python 3.6 as the tool of development.; and in experimentation and performance valuation, experimental data include Iris [68], Wine [68], and Yeast [68] and KDDCUP99 [69] data usually used in other studies for performance comparison and assessment. In this paper, data were divided into multidimensional data-based small and large-scale data to conduct the performance evaluation. Iris and Wine data are used in an experiment with the altered K-means algorithm. The Iris datasets used in the experimentation of the altered K-means algorithm have three classes (setosa, versicolor, and viginica) and four attributes. The Wine datasets have three classes (1, 2, and 3) and 13 attributes. The Yeast datasets have 9 attributes. Of a total of 47,112 samples, approximately 5436 samples and 78 attributed volumes of Iris, Wine, and Yeast objects were used in the study. For this study, the UCI open datasets were used, and the collected data (i.e., Wine, Iris, and Yeast data) were expanded by 26-fold each time, and finally, 5436 necessary data were selected after reprocessing them. The large-scale data are based on multidimensional data, utilizing the Blobs data and KDDCUP99 data provided by Scikit-learn. The KDDCUP99 data belong to the Python Feach family, which has 34-dimensional 4,898,431 samples and 42 attributes. It was utilized in the DARPA intrusion detection system in 1998. The dataset is classified into three groups whose scope is divided into basic, traffic, and content features. In this study, data whose scope is traffic features were utilized in the performance evaluation.

### 4.2. The Data and Procedure for Algorithm Verification

In this section, the Proposed Reinforce K-means algorithm (PKM) was compared through experiments with regard to PKM and MAK by utilizing Iris, Wine, Yeast, and the KDDCUP99 dataset provided by Scikit-learn. We conducted a performance evaluation in this section, focusing on the performance verification regarding the previously proposed algorithm problems. In this section, verification of the optimal number of clusters through principal component analysis, proof of optimized convergence, and data classification accuracy according to the selection of an initial centroid was performed. For this, we performed two types of experiments. Both types had the following conditions in the experiment: the maximum number of repeats was 100 or smaller, and the difference in square error was < 0.01%. In addition, the center point was flexibly selected in the existing K-means algorithm by utilizing DB, Silhouette, and SSE, and the initial center point was selected in the proposed algorithm PKM by applying outliers and a space partitioning model. First, the cluster correct classification rate, execution cost, and outliers were verified with regard to multi-dimensional small-scale data using the Iris, Wine, and Yeast datasets to compare and evaluate the performances of PKM and existing K-means algorithm. Second, the cluster validity, F-measure, and execution cost in relation to the initial k were verified with regard to multidimensional large-scale data by utilizing the KDDCUP99 data provided by Scikit-learn for the comparison and evaluation of performances of PKM and existing K-means algorithm. Furthermore, the data applied was divided into two scopes. In a clustering experiment of data included in performance evaluation as small data, a random center point of proximity scale should be utilized and applied to the choice of initial center point. The data distribution scope should be within 95% for data distance in standard normal distribution with a standard error rate of ± 3%. In a clustering experiment of data included in performance evaluation as large-scale data, a standard error rate of ± 3% should be applied for data distance in standard normal distribution applied to the selection of an initial center point.

### 4.3. Experiments and Performance Evaluation in Small Data

Reduction of dimensions and predicted outlier variables for multi-dimensional data were used to assess the proposed PKM and its counterparts in previous studies. Four criteria were applied to performance evaluation. First, the accurate classification rate will be measured to check the reliability of classification results of input data. Second, time complexity will be measured to measure the complexity of the proposed algorithm compared with the ones in previous studies. Third, the implementation costs of the K-means algorithm will be measured to compare and analyze its classification costs with those of the old algorithms. The implementation costs will be measured differently according to K. Fourth, the frequency of outliers will be measured, including the input data.

#### 4.3.1. Clustering Classification Ratio

**Figure 9:**
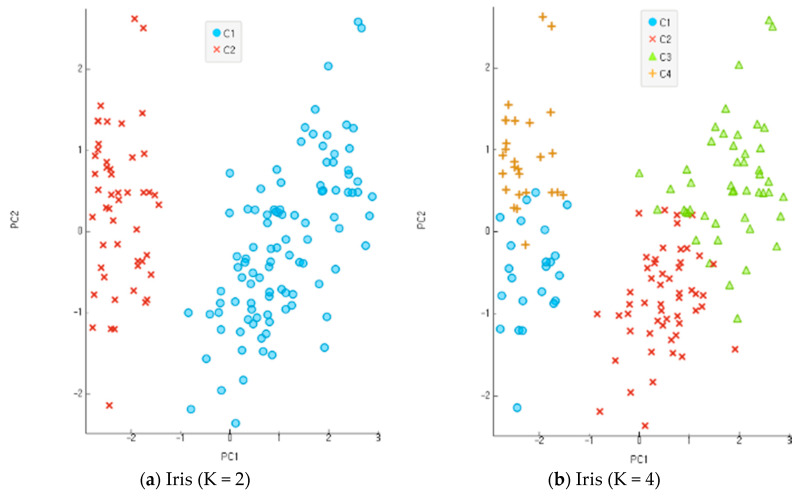

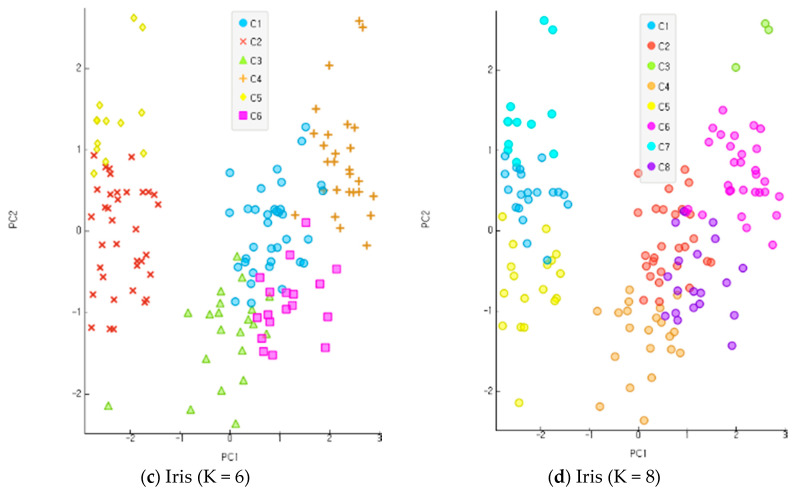
Experimentation results for each dataset when K, the number of clusters, was 2, 4, 6, and 8 in Iris data.

**Figure 10:**
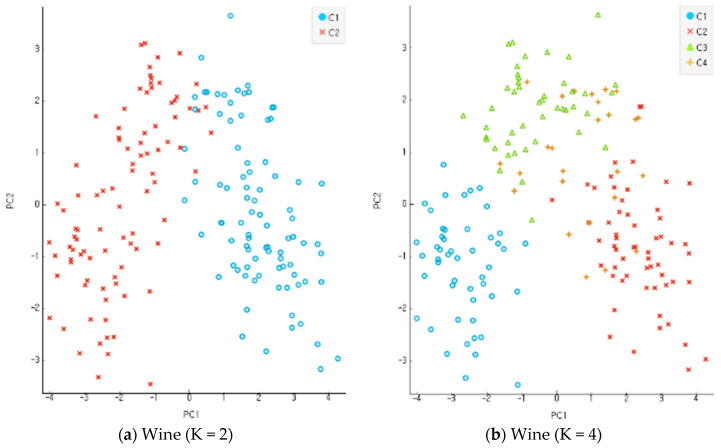

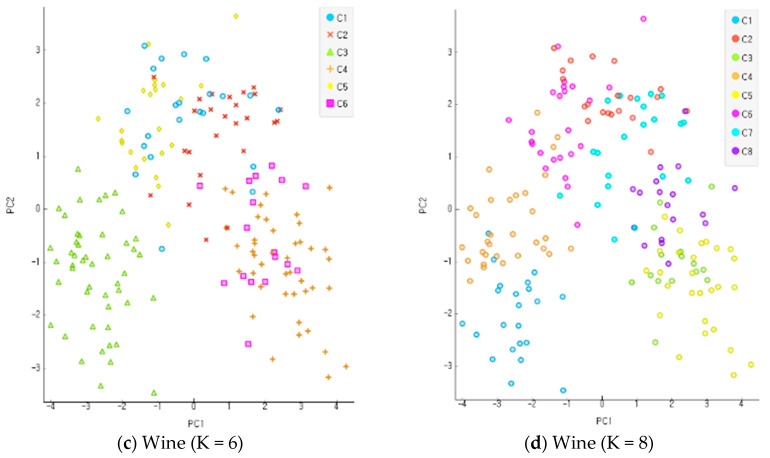
Experimentation results for each dataset when K, the number of clusters, was 2, 4, 6, and 8 in Wine data.

**Figure 11:**
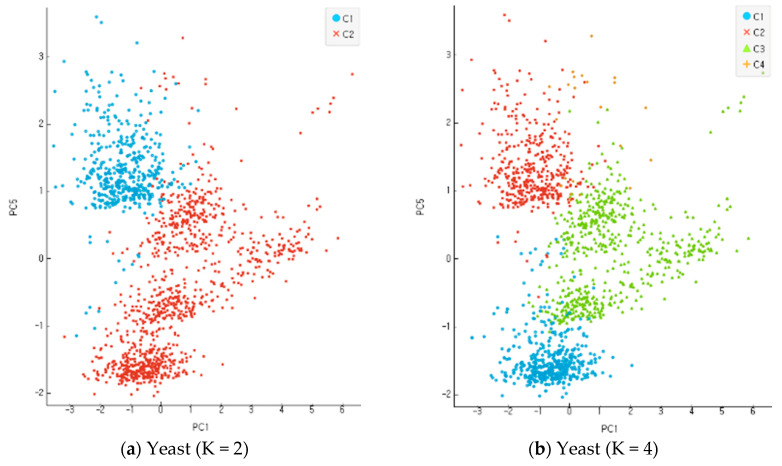

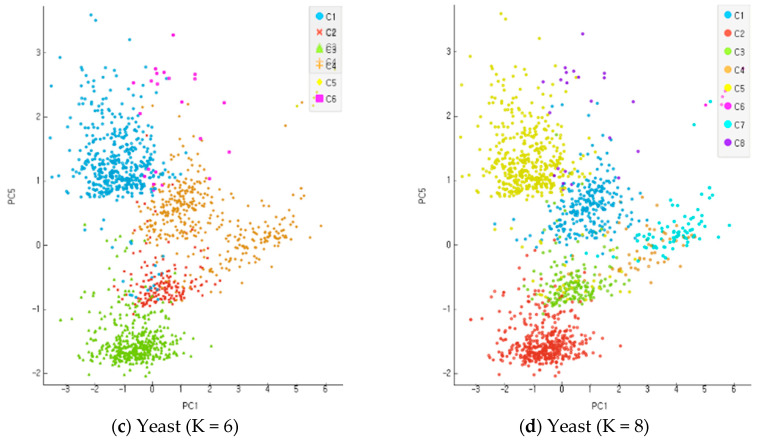
Experimentation results for each dataset when K, the number of clusters, was 2, 4, 6, and 8 in Yeast data.

In the present study, all the objects were used based on the re-substitution rule to obtain discriminants and calculate correct classification rates. A correct classification rate is obtained by dividing the number of misclassified objects with the total number of objects. The accuracy of clustering is assessed based on the differential levels of reliability for multi-dimensional data. An incompletion ratio makes an increment by 5% for each stage of evaluation data in the range of 10~30% with random virtual clustering outcomes in five stages. In the clustering process before the classification and analysis stage, the number of clusters increases from 2 to 8 in four stages for evaluation. Figure 9, Figure 10 and Figure 11 show the results of an experiment with four cases of K, the number of clusters, including 2, 4, 6, and 8 for each data set. Data clustering was conducted 150 times for each of the reliability levels. When data had higher reliability with the number of clusters within the scope of 2~4, the correct classification rate of the entire data was high. It also shows the application results of the altered K-means algorithm for each dataset. The Iris and Wine data are included in the range of K = 2–4, and the Yeast data are included in the range of K = 6–9. The findings indicate that the higher the reliability of the data, the higher the accurate classification rate of the entire data. Compared with the model of MAK [27] that did not conduct principal component analysis, the proposed model recorded higher accuracy for the classification results of the entire data, including the old data and error data. As seen in Table 1, the accurate classification rate tends to rise according to smaller numbers of clusters for Iris and Wine data and according to bigger number of clusters for Yeast data. The results of Table 1 have something to do with the optimal number of clusters through the principal component analysis of each dataset. The accurate classification rate rises according to the smaller number of clusters, because the number of objects classified as a different cluster from the initial one drops relatively in the process of assigning the objects to a cluster of high response rate after their clustering. In addition, the problems may lie in the formation of data rather than the model of clustering and analysis when the accurate and error classification rates are low and high, respectively. The causes are found in many error data being entered and distributed in the formation of initial data.

#### 4.3.2. Clustering Time Complexity

The model proposed in the study needs a calculation process of using principal component analysis and outliers compared with the approaches of initialization in the old K-means clustering. Thus, it is needed to interpret relations between time and input functions to treat the K-means algorithm in order to compare its performance with that of previous studies. Equation (9) shows the time complexity of the entire K-means algorithm.

(9) $$\begin{array}{ll} Time\;Complexit\\ & =T\left(Initial\;Cluster\;K\right)+T\left(Initial\;Centroid\right)\\ & +T\left(Assignment\;Recalculation\right) \end{array}$$

O(kn), the time complexity of the proposed algorithm, takes two major forms: T(k), the time complexity to process multidimensional data reduction, and T(2k), time complexity to calculate anticipated outliers and their cluster centroids according to the scatter plot vector. T(k), which represents time complexity to assign basic objects to their clusters and recalculate the centroids, is repeated (i), which means an increase by T(ik). The time complexity of the proposed algorithm will be eventually O(n) in Equation (10).

(10) $$\begin{array}{c} PKMTC=O\left(kn\right)+O\left(2kn\right)+O\left(ikn\right)\\ =O\left(n\right) \end{array}$$

#### 4.3.3. Clustering Performance Time (Cost)

The implementation costs of the proposed PKM algorithm were measured with multidimensional datasets: Iris, Wine, and Yeast. The measurement of implementation costs spanned from the entry of multidimensional datasets to the completion of final clustering. It was repeated 150 times to measure the mean time when K was 2, 3, 5 and 7. It was also compared and evaluated from those of previous studies to check its performance additionally.

**Figure 12:**
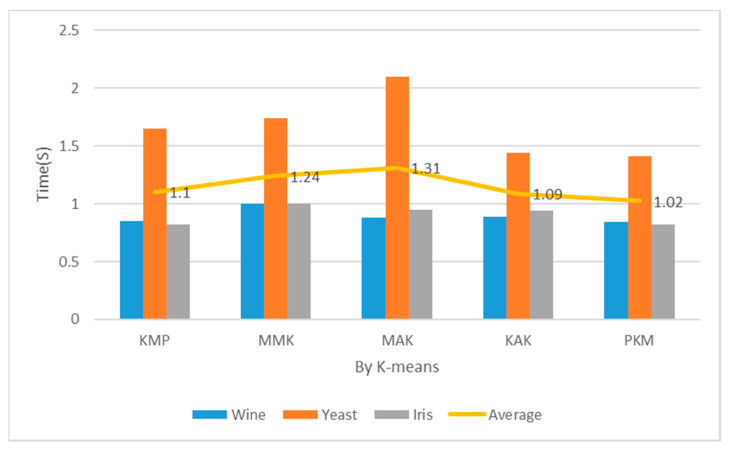
Mean performance cost (K = 2, Iteration = 150).

Table 2 and Figure 12 show the measurement results of implementation costs when the number of clusters was 2. In case of K = 2, the proposed algorithm recorded considerably lower implementation costs of clustering across all the datasets than KMP, MMK, MAK, and KAK. In case of clustering Wine and Iris datasets, the proposed algorithm recorded similar implementation costs to the KMP algorithm using distance and density and faster implementation costs by about 1–18% than the other algorithms. In case of Yeast datasets, the proposed algorithm recorded faster implementation costs by 33% or more than the MAK algorithm with concise time complexity. When the number of clusters was 2, the proposed algorithm reduced the implementation costs more than the KMP, MMK, MAK, and KAK algorithms by 7%, 17%, 22%, and 6%, respectively.

**Figure 13:**
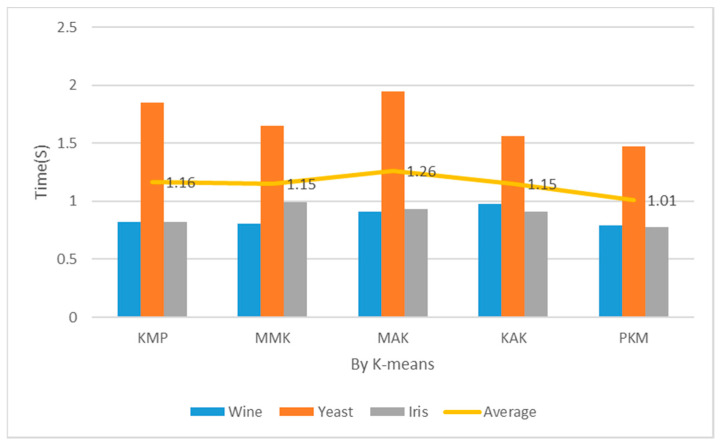
Mean performance cost (K = 3, Iteration = 150).

Table 2 and Figure 13 show the measurements of implementation costs when the number of clusters was 3. The overall results are similar to those of performance evaluation when K = 2, but the implementation cost assessment results of the Wine and Iris datasets were higher by 3% each. The partial reasons were the choice of optimal K = 3 through principal component analysis and the reduced final implementation costs of clustering according to the optimal number of clusters. When the number of clusters was 3, the proposed algorithm reduced the implementation costs more than the KMP, MMK, MAK, and KAK algorithms by 13%, 12%, 20%, and 12%, respectively.

**Figure 14:**
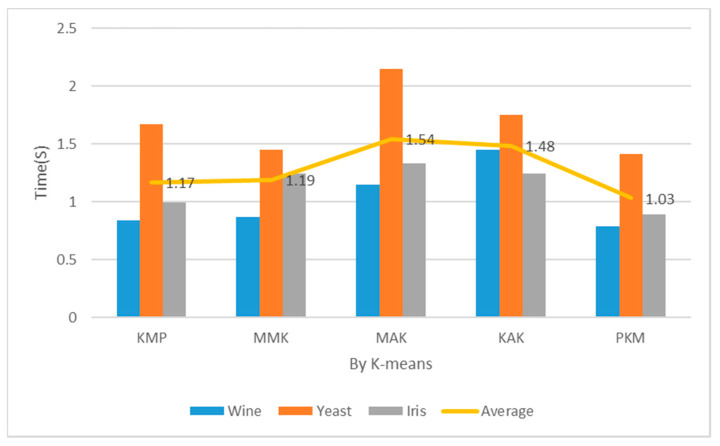
Mean performance cost (K = 5, Iteration = 150).

**Figure 15:**
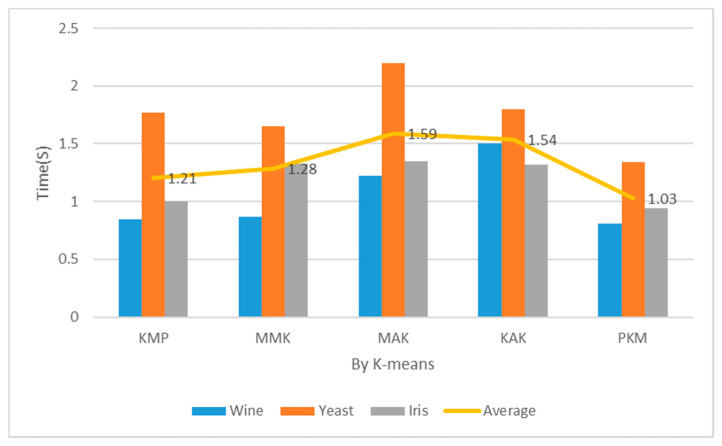
Mean performance cost (K = 7, Iteration = 150).

Table 2 and Figure 14 show the implementation costs results when the number of clusters was 5. In addition, Table 2 and Figure 15 show the implementation costs results when the number of clusters was 7. When K was 5 and 7 for the Iris datasets, the proposed algorithm reduced the implementation costs by an average of 24.5% more than the other algorithms. When the optimal number of clusters was 7 for the Yeast datasets through principal component analysis, it reduced the implementation costs by an average of 27% more than the other algorithms. When the number of clusters was 5, the proposed algorithm reduced the implementation costs more than the KMP, MMK, MAK, and KAK algorithms by 12%, 13%, 33%, and 30%, respectively. When the number of clusters was 7, it reduced the implementation costs more than the KMP, MMK, MAK, and KAK algorithms by 15%, 20%, 35%, and 33%, respectively.

#### 4.3.4. Clustering Performance of Outlier Detection

**Figure 16:**
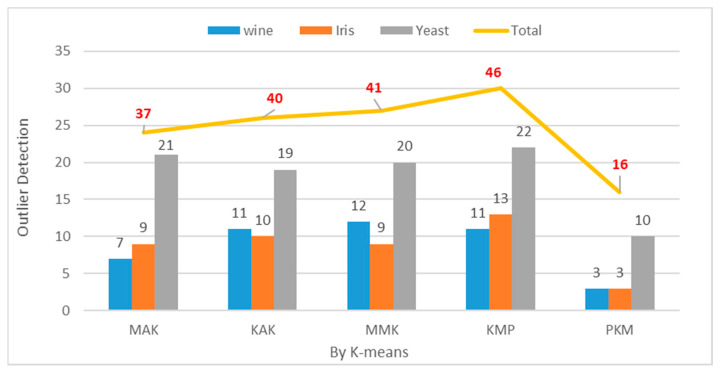
Outlier Detection by Data Set ((K = 3, 5, 7), Iteration = 150).

When objects are included in a cluster containing three objects or less or record a considerably smaller dissimilarity distance from the cluster centroid or cohesion with the internal objects, they will be classified as outliers. In the performance experiment, clustering was repeated 150 times for the Wine, Iris, and Yeast datasets when the optimal number of clusters K was 3, 5, and 7. Table 3 and Figure 16 show the average frequency of outliers for each dataset between the old algorithms and the proposed one, which lowered the frequency of outliers to a great degree by using the initial cluster centroids as anticipated outliers and the object with the longest distance from the initial centroid as the second centroid. The production rate of outliers decreased by approximately 69% when the number of clusters was 3 in the Wine datasets. It decreased by approximately 70% when the number of clusters was 3 in the Iris datasets. It decreased by approximately 51% when the number of clusters was 7 in the Yeast datasets.

### 4.4. Experiments and Performance Evaluation in Large Data

**Figure 17:**
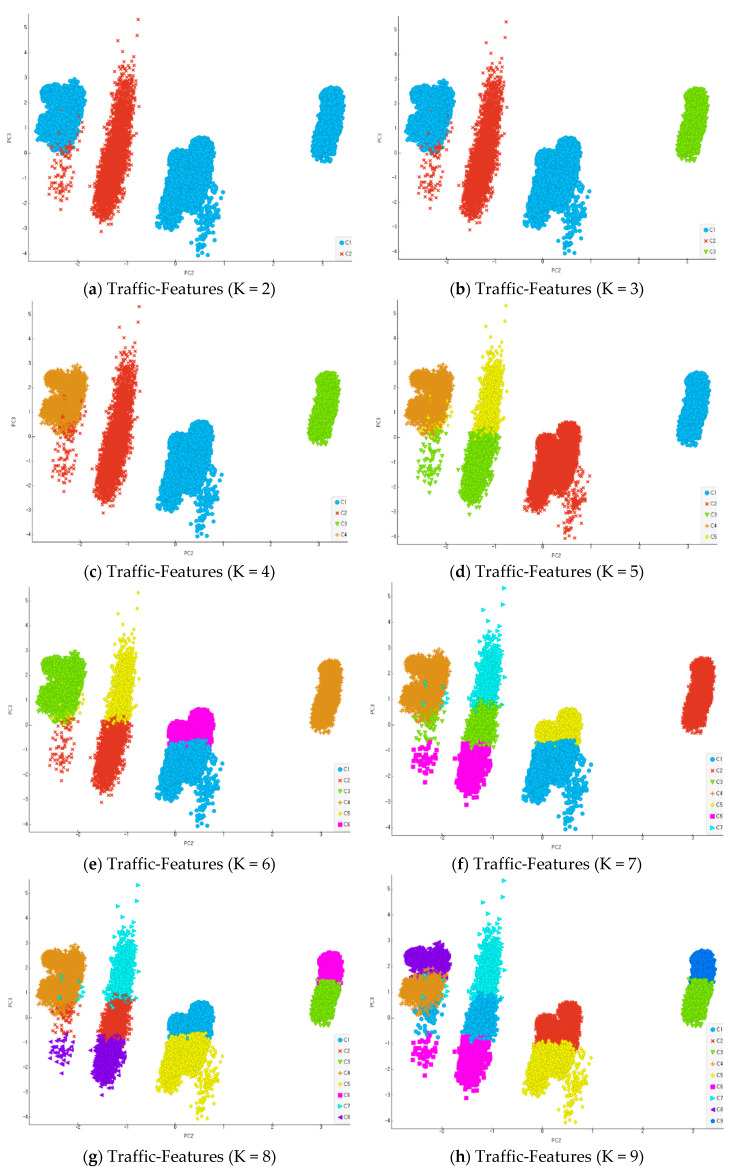
Clustering by data classification (KDDCUP99-Traffic Features).

In this section, experiments employing a large-scale dataset were conducted to apply the Proposed Reinforce K-means algorithm (PKM) to large data. The reduction in multidimensional data dimension and prognostic variables of outliers were utilized to improve the accuracy of clustering results in the experiments. For the performance evaluation of the K-means initialization approach algorithm, two measures were used: the clustering validity measure of the proposed algorithm that can evaluate the cohesion and separation between clusters compared to those of the existing K-means algorithm, and the F-measure, which can evaluate the accuracy of clustering. In particular, since the K-means algorithm is effective in the non-labeled data classification of large data, cluster validity through the selection of an automated K value and initial center point, which is the goal of this study, is a very important measure. Performance evaluation was conducted utilizing 250,458 data records (prior labeling processed) out of 18-dimension traffic features data from the KDDCUP99 dataset previously utilized in the performance evaluation. Figure 17 shows the results when the K value is 2 as the minimum value of clustering, up to 9.

#### 4.4.1. Performance Evaluation of CVI

**Figure 18:**
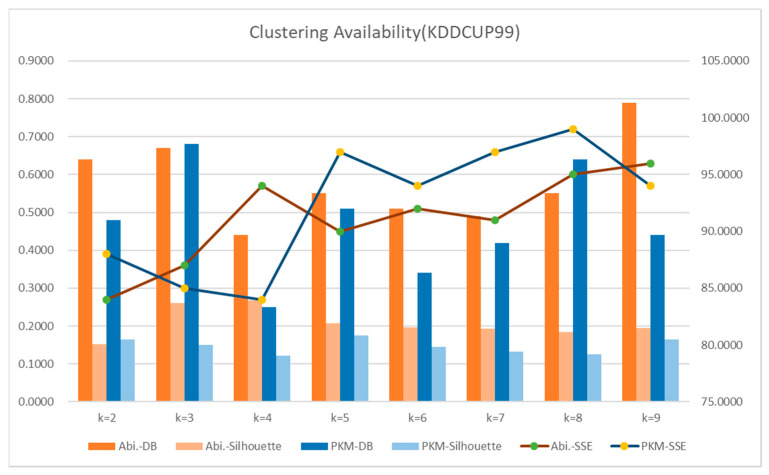
Clustering by data classification (KDDCUP99-Traffic Features).

In the experiment, a K value from 2 to 9 was used to verify the cluster validity by K value, i.e., the optimized scope of cluster K value. The existing K-means algorithm and the PKM (Proposed Reinforcement K-means) algorithm proposed in this study were compared by using the Davies–Bouldin (DB) model, Silhouette, and Sum of Squared Errors (SSE) to determine the validity with regard to the optimized K value. The reliability of PKM proposed in the study was measured with CVI (Clustering aVailability Index), a measuring model to divide the total sum of inter-cluster cohesion based on individuals and centroids of clusters with the separation between centroids of clusters. The measurements show that K, the minimum value, could be determined as an optimal number of clusters. It followed the old research approach of deciding K based on the DB model to measure distance, the silhouette method, and the comparison and analysis of SSE measurements. A DB model metric is the addition of cohesion among clusters based on the objects and center points of a cluster divided by the separation between the center points of the cluster. The scope of the minimum value can be selected as an optimal value K. A silhouette metric offers a criterion to measure the coherence of data grouping within clustering. A silhouette coefficient is between −1 and 1. When the separation force of clustering equals the cohesion force of clustering, the silhouette coefficient is 0. An SSE metric measures the distance between each data and their adjacent clusters. When SSE differences are smaller between clusters, they can be defined as an optimal number of clustering. The DB model, Silhouette, and SSE as the evaluation techniques used in the experiment were repeatedly executed 150 times for each section, and optimized results of Silhouette (0.1223) and SSE (84.000) were found when K was 3 through the proposed K-means algorithm (PKM). Good clusters were derived if the K value was selected when the slope was lower compared to the number of the next cluster in SSE. As measurement models conducted in the existing K-means algorithm (Abi.) [64], Silhouette (0.1513) had K = 2 and SSE (91.00) had K = 7, which selected extremely different values, thus giving rise to problems of high probability of outlier occurrence and many duplicate objects in a cluster. In the existing K-means algorithm, the K value selection through the DB model derived the same results as those of our study results. When K was 3, the duplicate data were removed, and the classification scope was clearly separated. To verify the optimized cluster results and accuracy of the previously labeled data, the accuracy was verified through F-measure. Figure 18 and Table 4 present the results that verify the cluster validity index of the classified results from the KDDCUP99 data.

#### 4.4.2. Performance Evaluation

Labeling was processed for the dataset before the F-measure measurement conducted in this section. Through such processing, the cluster data were analyzed to determine the accuracy of the clustering results regarding the dataset and different accuracy values were measured depending on whether the correct cluster data were present or not based on the clustering results. The accuracy was measured by F-measure, which was the most widely used to measure the accuracy of clustering in terms of clustering results for the implementation of the K-means algorithm. Clustering was conducted in advance for data for performance evaluation. The experiments were performed using 50,000 data records in the KDDCUP99 dataset used in the performance evaluation. The first group collects only the connections for the past two seconds that have the same host function and same destination host as those of the current connection. This group contains data in relation to protocol behavior and service, etc. The second group has data that inspect only the connections for the past two seconds with the same service as that of the current connection. These two groups are categorized into nine areas: count, serror rate, rerror rate, same srv rate, doft srv rate, srv count, srv serror rate, srv rerror rate, and srv dift host rate. Here, 50,000 records of the count data that collect the connections with the same destination host as that of the current connection from the previously classified data are verified. The detailed criteria of the accuracy measurement were defined by the clusters of the dataset as follows: true positive (TP), true negative (TN), and false positive (FP). More specifically, TP refers to a case wherein the clustered object in Cluster 2 is also clustered in Cluster 2 after the experiment. TN refers to a case wherein the clustered object in Cluster 2 is not clustered in Cluster 2 after the experiment. FP refers to a case wherein objects that are not clustered in Cluster 2 are clustered in Cluster 2 after the experiment. Table 5 presents the measurement results of clustering. The performance evaluations were conducted based on a K value of 4, which was optimized through the PKM algorithm, and the K values 2, 3, and 7, which were selected through the existing K-means algorithm. Nonetheless, evaluations may deviate when K was 2. Thus, the K 2 scope was excluded, and evaluations were conducted with the scopes of the rest, i.e., 3, 4, and 7. As presented in Table 5, when PKM was applied to the KDDCUP99 data, the performance evaluation results showed an average precision rate of 94.61% and a recall rate of 96.91% when K values 3, 4, and 7 were applied during the classification of 50,000 records of count data. F-measure was 95.30% based on the data precision and recall rates. In particular, the precision, recall rates, and F-measure results were all higher in a scope classified with the optimized K value. When the K value was 7, the TN and FP values were relatively higher due to the duplicate arrangement between objects. These performance evaluation results demonstrate that the proposed algorithm guarantees higher precision for data clustering. In case of applying the proposed algorithm along with accuracy rates, another round of performance evaluation would be added to identify and assess errors in the given dataset. The sizes and values of error data were added according to the scope of artificial datasets to detect error data. In case of KDDCUP99, the integers sampled from a set {r: 1500 < r < 2000} were added for errors added to each characteristic. A total of 10 of the entire datasets were used in evaluation. Two standard datasets and four error datasets were added to the standard dataset by 0%, 5%, 10%, 15%, and 20%. A total of 10 datasets were used including two containing no outliers and eight containing an outlier. Table 6 shows the results of 100 performance evaluations for the KDDCUP99 dataset and the comparison outcomes of mean errors. Each algorithm had 50 repetitions. Datasets to be applied to the existing clustering initial approach algorithm [64] and the proposed clustering algorithm were applied the same randomly within the dataset scope to assess the proposed initialization selection program after 100 experiments. The clustering K value was set at 4 to ensure the accuracy of evaluation. As seen in Table 6, when the same K value was applied, mean errors according to an initialization choice were lower in the proposed algorithm than the old clustering technique across all the datasets in the experiment. These findings indicate that the proposed algorithm had superior performance to the old clustering algorithm in the clustering of outliers instead of inliers at a random initial center point. Unlike the proposed algorithm, the old clustering algorithm was not able to tell outliers from inliers and carried out clustering only after the choice of initial center point.

## 5. Conclusions

Many of the previous studies on the K-means algorithm selected an initial centroid arbitrarily, used it to measure the similarity of an object to other objects, and assigned K, the number of clusters, accordingly. Most of the K-means researchers have pointed out a disadvantage of outliers belonging to external or other clusters instead of the concerned ones when K is big or small. Thus, the present study analyzed problems with the selection of initial centroids in the old K-means algorithm and investigated a new K-means algorithm of selecting initial centroids. In addition to the algorithm [66] of selecting K, the optimal number of clusters, in previous studies, the present study proposed another algorithm of selecting an initial centroid of a cluster through the outliers and spatial division of classification data capable of producing ideal clustering outcomes. In previous studies on clustering, users would select an initial centroid arbitrarily or use a randomly selected individual to measure its similarity to other individuals. The algorithm proposed in the present study proceeded with clustering by choosing an outlier individual reflecting negative impacts on clustering as an initial centroid of clustering. An individual within the outlier scope on its standard normal distribution was used as an initial centroid in the proposed method. For the selection of outlier objects, the objects in the range of outliers in the standard normal distribution of clustering objects were used as initial centroids. The findings demonstrate the effects of reducing the overall clustering calculation costs in the proposed algorithm that was not dependent on the initial cluster centroid and made use of the lower dependency between objects. The performance experiment results of the proposed algorithm show that it lowered the execution costs by about 13–14% from those of previous studies (MAK, KAK, MMK, KMP) when there was an increase in the volume of clustering data or the number of clusters. It also recorded a lower frequency of outliers, a lower effectiveness index, which assesses performance deterioration with outliers, and a reduction of outliers by about 60%.

An improved algorithm for selecting an initial centroid for a K-means algorithm was proposed in this study in an attempt to enhance the data classification accuracy and performance for the future studies. It is expected that the analyzing or predicting of various types of structured data will be improved in terms of ‘performance’ compared to the existing studies.

As a future work, the optimized K-value algorithm proposed in the previous paper [66] and the initial centroid approach algorithm based on ideal points and space division proposed in this paper were established to conduct performance evaluation. However, if there are dense data in the optimal K-value measurement and initial centroid approach selection, it will be necessary to make up for the fact that there is an increase in the misclassification rate or a mis-selection of the initial centroid approach in the stochastic space measurement.

## Figures and Tables

**Table 1:** Clustering Classification Ratio. MAK: Macqueen Approach K-means.

Reliability	Dataset	Existing Model (MAK)	Proposed Reinforcement K-Means Algorithm (PKM)				
			Average	K = 2	K = 4	K = 6	K = 8
90%	Wine	0.9663	0.9874	0.9944	0.9944	0.9831	0.9775
	Yeast	0.9461	0.9618	0.9596	0.9677	0.9589	0.9609
	Iris	0.9667	0.9867	0.9933	0.9933	0.9867	0.9733
	Average			0.9824	0.9851	0.9762	0.9706
85%	Wine	0.9551	0.9635	0.9888	0.9831	0.9382	0.9438
	Yeast	0.9124	0.9532	0.9555	0.9319	0.9555	0.9697
	Iris	0.9467	0.9700	0.9733	0.9867	0.9600	0.9600
	Average			0.9725	0.9673	0.9512	0.9578
80%	Wine	0.9494	0.9438	0.9438	0.98788	0.9663	0.9382
	Yeast	0.8484	0.9313	0.9319	0.9340	0.9171	0.9420
	Iris	0.9333	0.9533	0.9600	0.9533	0.9600	0.9400
	Average			0.9453	0.9587	0.9478	0.9401
75%	Wine	0.8820	0.9185	0.9270	0.9326	0.9213	0.8933
	Yeast	0.8248	0.8972	0.8962	0.8747	0.8868	0.9313
	Iris	0.8800	0.9033	0.9133	0.9200	0.9067	0.8733
	Average			0.9122	0.9091	0.9049	0.8993
70%	Wine	0.7528	0.8708	0.8820	0.9101	0.8315	0.8596
	Yeast	0.7951	0.8624	0.8726	0.8315	0.8336	0.9117
	Iris	0.8400	0.8900	0.9133	0.8800	0.8933	0.8733
	Average			0.8893	0.8739	0.8528	0.8815

**Table 2:** Mean Performance Cost (Iteration = 150). KAK: Kaufman Approach K-means, MMK: Max–Min Approach K-means.

Cluster	Item	Wine (s)	Yeast (s)	Iris (s)	Avg. (s)
2	KMP	0.85	1.65	0.82	1.10
	MMK	1.00	1.74	1.00	1.24
	MAK	0.88	2.10	0.95	1.31
	KAK	0.89	1.44	0.94	1.09
	PKM	0.84	1.41	0.82	1.02
3	KMP	0.82	1.85	0.82	1.16
	MMK	0.81	1.65	0.99	1.15
	MAK	0.91	1.95	0.93	1.26
	KAK	0.98	1.56	0.91	1.15
	PKM	0.79	1.74	0.78	1.01
5	KMP	0.84	1.67	0.99	1.17
	MMK	0.87	1.45	1.24	1.19
	MAK	1.15	2.15	1.33	1.54
	KAK	1.45	1.75	1.24	1.48
	PKM	0.79	1.41	1.89	1.03
7	KMP	0.85	1.77	1.00	1.21
	MMK	0.87	1.65	1.33	1.28
	MAK	1.22	2.20	1.35	1.59
	KAK	1.50	1.80	1.32	1.54
	PKM	0.81	1.34	0.94	1.03

**Table 3:** Mean Performance of Outlier Detection (Iteration = 150).

Item	Wine (K = 3)	Yeast (K = 7)	Iris (K = 3)	Total
KMP	11	20	13	46
MMK	12	20	9	41
MAK	7	21	9	37
KAK	11	19	10	40
PKM	3	10	3	16

**Table 4:** Experiment of Clustering Availability Index (Iteration = 10). DB: Davies–Bouldin, SSE: Sum of Squared Errors.

Item	K = 2	K = 3	K = 4	K = 5	K = 6	K = 7	K = 8	K = 9
Abi.-DB	0.6400	0.670	0.400	0.5500	0.5100	0.4900	0.5500	0.7900
Abi.-Shilhoutte	0.1513	0.2609	0.2683	0.2073	0.1964	0.1931	0.1847	0.1945
Abi.-SSE	84.0000	87.0000	94.0000	90.0000	92.0000	91.0000	95.0000	96.0000
PKM-DB	0.4800	0.6800	0.2500	0.5100	0.3400	0.4200	0.6400	0.4400
PKM-Shiloutte	0.1654	0.1510	0.1223	0.1744	0.1451	0.1324	0.1247	0.1642
PKM-SSE	88.000	85.0000	84.0000	97.0000	94.0000	97.0000	99.0000	94.0000

**Table 5:** Clustering by Data Classification (Iteration = 10).

Cluster	Object	TP	TN	FP	Precision	Recall	F-Measure
3	50,000	30,000	914	747	97.04	97.57	97.31
4	50,000	30,000	874	642	97.17	97.90	97.54
7	50,000	30,000	3476	2415	89.62	92.06	91.06
Avg.	50,000	30,000	1755	1268	94.61	96.01	95.30

**Table 6:** Comparing Errors of Proposed Reinforcement K-Means.

Dataset (with Outlier Rate in %)	Mean Square Error	
	Abi.	PKM
KDDCUP99 (0%)	853.21	614.45
KDDCUP99 (5%)	1847.79	1518.16
KDDCUP99 (10%)	1974.45	1644.55
KDDCUP99 (15%)	2674.84	1879.68
KDDCUP99 (20%)	3074.58	2378.17

## Abbreviations

IntraCD	Intra-Cluster Distance
ICD	Inter-Cluster Distance
AIC	Akaike Information Criterion
BIC	Bayesian Inference Criterion
MAK	Macqueen Approach K-means
KAK	Kaufman Approach K-means
MMK	Max-min Approach K-means
AIC	Akaike Information Criterion
BIC	Bayesian Inference Criterion
MLE	Maximum Likelihood Estimate
